# Epidemiological Study of Adamantinoma from US Surveillance, Epidemiology, and End Results Program: III Retrospective Analysis

**DOI:** 10.1155/2020/2809647

**Published:** 2020-06-16

**Authors:** Mahmut Nedim Aytekin, Recep Öztürk, Kamil Amer

**Affiliations:** ^1^Department of Orthopedics and Traumatology, Ankara Yildirim Beyazit University, Ankara, Turkey; ^2^Department of Orthopedics and Traumatology, Dr Abdurrahman Yurtaslan Oncology Training and Research Hospital, Ankara 06200, Turkey; ^3^Department of Orthopaedics, Rutgers New Jersey Medical School, 140 Bergen Street, Suite D, Newark, NJ 07103, USA

## Abstract

**Objective:**

Adamantinomas are rare low-grade malignant bone tumors. This study aims to describe the demographic characteristics and survival rates of patients suffering from adamantinomas.

**Methods:**

The National Institute of Cancer Surveillance, Epidemiology, and Recent Results (SEER) database was used, and patients diagnosed with adamantinoma between 1973 and 2016 were screened. Patients were classified according to sex, age, race/ethnicity, and marital status, and also tumors were classified according to year of diagnosis, laterality, type of treatment, and follow-up.

**Results:**

The mean age of patients was 30.8 ± 16.7 (range: 4–75). A total of 92 patients were identified; of these, 43 were females and 49 were males. The mean follow-up period was 138.1 ± 90.3 (range: 1–156) months. Mean survival duration was 287.8 ± 15.4 (95% CI: 257.7–317.9) months. Five- and ten-year survival rates were 98.8% and 91.5%, respectively. Besides, survival time was also observed to be independent of gender, age groups, race, marital status, tumor location, and year of diagnosis.

**Conclusion:**

Adamantinoma is a very rare bone tumor that affects the long bones in lower extremities and is more common in men. Five- and 10-year survival prognoses are reasonably satisfactory. Also, survival time is independent of variables such as gender, age, and tumor location.

## 1. Background

Adamantinomas are low-grade malignant neoplasms characterized microscopically by clusters of epithelial cells surrounded by a light spindle cell sheath [1]. It is a rare tumor that accounts for less than 1% of all primary bone tumors [2].

The most common age range for occurrence is between 20 and 50 years, and it is more common in men [3, 4]. Tibia is the most affected area (85% of reports) [5].

Pain is the main symptom reported by the patients [6, 7]. X-ray usually shows an aggressive pattern, and a poorly sclerotic lesion with radiolucent areas is seen [8]. Adamantinoma treatment involves extensive resection of the lesion and reconstruction of bone defect. Considering adamantinomas are low-grade tumors, radical surgical interventions such as amputation should be avoided as much as possible [9]. Several procedures including autograft, allograft, bone transport, and endoprosthesis have been described for reconstruction [8]. Chemotherapy or radiotherapy has no place in treatment, and local recurrence and, furthermore, distant metastasis are rare, and if metastasis develops, it is most commonly in lungs [4, 10].

To the best of our knowledge, this is the first study analyzing the epidemiology and survival rates of adamantinoma from the SEER database. We aimed to define demographic characteristics of adamantinoma, its incidence, and survival rates.

## 2. Methods

A retrospective review of adamantinoma cases was performed using the latest version of the Surveillance, Epidemiology, and End Results (SEER) database published in November 2018. This release covers the years between 1975 and 2016. The SEER database is the most complete and comprehensive registry of cancer incidence and survival data in the United States. [11] Previously, it has been approved and used in cancer studies for many surgical subspecialties. Since 1973, the SEER database has been compiling cancer data collected from public registry records and now represents 28% of the US population [11, 12]. The SEER database is mainly used in study of rare diseases such as adamantinomas; it provides extensive multi-institutional data to increase the representative and statistical power of study, which is not possible to replicate with other options.

For this study, data were surveyed with “adamantinoma of long bones” (ICD-O-3 code number 9261/3) code in the International Classification of Diseases for Oncology, Third Edition (ICD-O 3), morphology code system [13]. Patients were classified according to gender, age, race/ethnicity, and marital status. Age data were represented in two categorical variables (greater than 30 years and less than or equal to 30 years). Tumors were classified according to the year of diagnosis, laterality, type of treatment, and follow-up duration. The year of diagnosis was divided into two groups labeled as “1973–1997″ and “1998 and beyond.” Follow-up results were evaluated in two groups as alive and deceased. Surgical treatment types were grouped as nothing, marginal excision, radical resection, amputation, and unknown. Margin status is not abstracted by SEER and thus could not be analyzed.

In the SEER database, the methodology for determining the cause of death is “Cause-specific Death Classification,” but the cause of death is difficult to associate and incorrect distributions are reported [14]. As a result, it is not clear whether SEER data accurately report deaths from illness or other causes.

All patient identifiers are omitted from SEER database data, and as a result, studies using the SEER database are exempt from Institutional Review Board approval.

Statistical analysis was performed by SPSS 22.0 (Chicago, IL) computer program. In statistical analysis, categorical variables were given as numbers and percentages, and continuous variables were presented with mean ± standard deviation (SD) and median (min-max value) for descriptive analyses. Survival analyses were performed by Kaplan–Meier methods and log-rank tests. *p* < 0.05 was considered to be statistically significant.

## 3. Results

**Figure 1:**
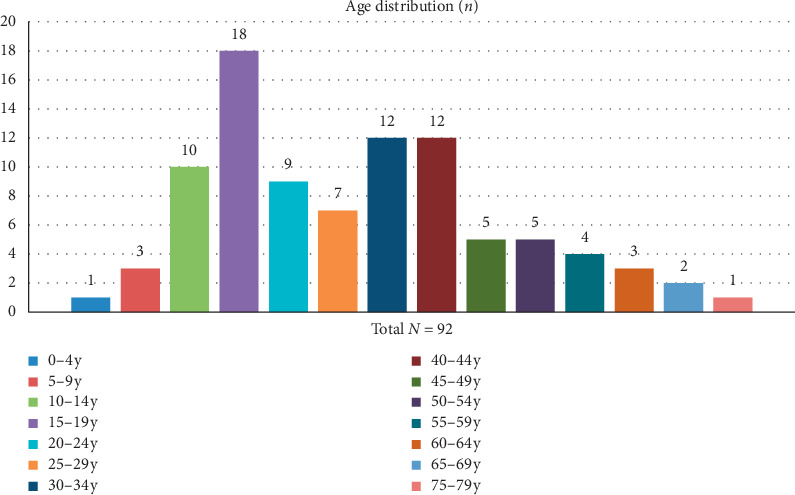
Age distribution of patients.

A total of 92 patients (mean age 30.8 ± 16.7 (range: 4–75); 43 females and 49 males) were included in our analysis of SEER data. 61.4% of patients included in this study were single, and 55.4% were under 30 years of age. Detailed age distribution is presented in Figure 1.

When lesion locations of the 92 patients were examined, it was found that 89 (96.7%) were located in the lower extremity long bones, 2 were located in the upper extremity long bones, and for one patient, lesion location was not registered.

The mean follow-up period was 138.1 ± 90.3 (range: 1–156) months. When racial characteristics were examined, it was observed that white race constituted 73.9% of patient population. In this study, 66 patients were diagnosed after 1998. 54.9% of tumors were located in right extremity. Seven patients had no surgical treatment, 22.8% underwent marginal excision, 54.3% went through radical resection, and 7.6% were amputated. Surgical treatment information of 7 patients in this study was not registered (Table 1).

**Figure 2:**
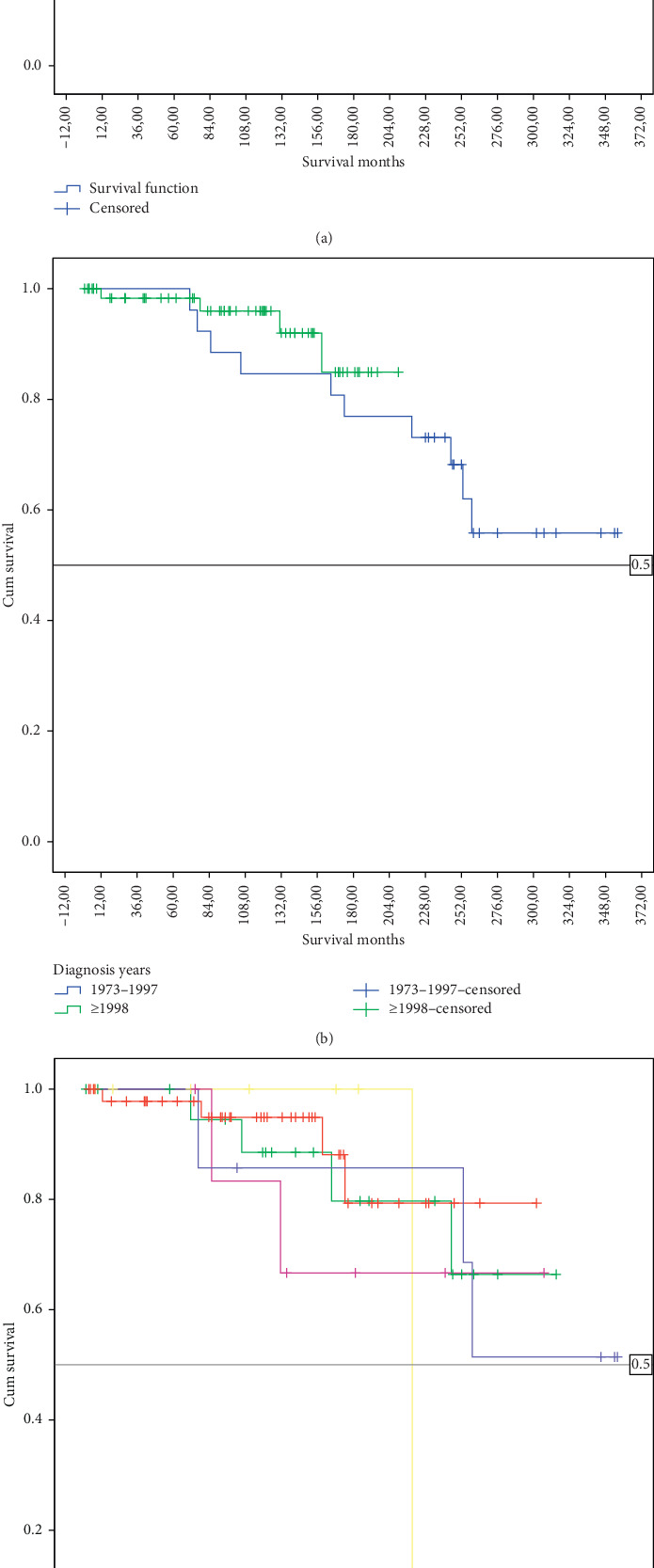
(a) Kaplan-Meier curve for overall Survival. (b). Kaplan-Meier curves for overall survival according to diagnosis years. (c). Kaplan-Meier curves for overall survival according to surgical treatments.

Of the 92 patients included in this study, 14 (15.2%) deceased. The mean survival time was 287.8 ± 15.4 (95% CI: 257.7–317.9) months. Median survival could not be determined in this study. The 5-year survival rate was 98.8%, and the 10-year survival rate was 91.5% (Figure 2(a)).

In addition to that, survival rates were found to be similar among gender (log-rank test; *p*=0.128), age groups (log-rank test; *p*=0.169), race (log-rank test; *p*=0.179), marital status (log-rank test; *p*=0.481), the side where the tumor settled (log-rank test; *p*=0.169), whether surgery is performed (log-rank test; *p*=0.809), and year of diagnosis (log-rank test; *p*=0.374) (Table 2). The 5- and 10-year survival rates of patients are presented in Table 2. The 10-year survival rate was 96% in patients diagnosed after 1998, while it was 84.6% in the ones diagnosed before 1998 (Figure 2(b)). The patient group received surgery has a 5- and 10-year survival rate of 98.6% and 91.5%, respectively. Additionally, a more in depth study of treatment subgroups revealed a 10-year survival rate of 94% in the radical resection group and 88.5% in the marginal excision group (Figure 2(c)).

## 4. Discussion

The main aim of this study was to determine the demographic characteristics, incidence, and survival rates of adamantinoma cases registered in the SEER database. This database allows for detailed examinations of large patient cohorts and rare tumors, including adamantinomas. Our study identified 92 patients diagnosed with adamantinoma between 1973 and 2016 in the SEER database.

Due to rarity of this disease in the literature, there is not much data related to adamantinoma. Most of the studies are in the form of case reports and retrospective analyses [1, 4–6]. For this study, we were able to find 92 patients registered in the database in the last 43 years.

As reported in literature, the survival rate of adamantinoma is excellent, with a 5-year survival rate of 85%–95% [15]. Ten-year survival rates are reported to be around 85% [6]. In our opinion, since this is a study involving a relatively large number of patients, it may provide more accurate information. In this study, the 5-year survival rate was found to be 98.8%.

There is controversy over whether OFD (osteofibrous dysplasia) is a precursor lesion to adamantinoma or whether it is a residual lesion left by spontaneously regressive adamantinoma [16, 17]. Also, a different clinical entity, called differentiated or OFD-like adamantinoma, was positioned between OFD and adamantinoma [17, 18]. While Scholfield et al. have reported no deaths on average of 10 years of follow-up in 42 OFD and 10 OFD-like adamantinoma cases, they have reported a 10-year survival as 93% in adamantinoma [16]. Insufficiency in determining whether the cause of death in the SEER database is due to illness or other causes, and the high survival rates in this extensive series used in this study may indicate that survival rates in the presence of this tumor do not differ from the average human survival.

The mean age in our study was 30.8 years, which was consistent with the literature [19]. In the literature, it is reported to be more common in men [4, 8]. Our study confirmed that as 53% of cases were men.

We did not provide any information about recurrence and complications since it was not included in our data. However, when the literature is examined, it can be seen that local recurrence rates between 18% and 32% are reported [4].

Adamantinoma is most commonly located in long tubular bones such as the tibia fibula and ulna and is rarely located in the femur humerus and radius [20–22]. In our data, at least 96.7% of the lesions were located in the long bones of the lower extremities.

In the literature, the preferred treatment in all cases is extensive resection of the tumor and reconstruction of the defective area [4, 9]. Positive surgical margins are associated with high local recurrence and metastasis in adamantinoma [15]. Qureshi et al. in their multicenter study, which retrospectively evaluated 70 cases, found no meaningful relationship between wide operative margins and survival. They linked this to the limited number of patients [9]. In that study, while 10-year survival was 94% in the radical resection (resection with wide margins) group, it was 88.5% in the marginal excision group.

There were some limitations to this study. First, it was a retrospective analysis, and although it included many years, the number of patients was relatively small, as the disease in question is rare.

Since this study focuses on database analysis, and detailed data for all patients in the database could not be found, some patients were not included and some of the demographic data and all results related to the disease were not mentioned. There are no data on the recurrence status of patients in the SEER database. Also, some important critical data about semimalignant tumors, such as metastasis data, cannot be reported based on SEER data. Recurrence and metastasis data of the patients were not available in this study. In addition, since some of the data were more general, detailed data could not be analyzed sufficiently. Extensive studies with better documentation and higher patient numbers are needed in the future.

## 5. Conclusion

This is the first adamantinoma study using the SEER database and is the most extensive adamantinoma series in the literature. Eventually, adamantinoma is a very rare bone tumor that affects the long bones of the lower extremities and is more common in men. The 5- and 10-year survival rates are very high. In addition, survival time is independent of gender, age group, race, marital status, tumor location, and year of diagnosis.

## Figures and Tables

**Table 1:** Basaline demographics.

Characteristic	Total *N* = 92
Age, year	
Mean ± sd	30.8 ± 16.7
Median (min-max)	27.5 (4.0–75.0)

Age, *n* (%)	
≤30	51 (55.4)
>30	41 (44.6)

Sex, *n* (%)	
Female	43 (46.7)
Male	49 (53.3)

Race, *n* (%)	
White	68 (73.9)
Black	10 (10.9)
Other	14 (15.2)

Marital status (*n* = 88), *n* (%)	
Single	54 (61.4)
Married	34 (38.6)

Laterality (*n* = 91), *n* (%)	
Left	41 (45.1)
Right	80 (54.9)

Diagnosis years, *n* (%)	
1973–1997	26 (28.3)
≥1998	66 (71.7)

Surgical treatment, *n* (%)	
Nothing	7 (7.6)
Amputation	7 (7.6)
Marginal excision	21 (22.8)
Radical resection	50 (54.3)
Unknown	7 (7.6)

Follow-up time, months	
Mean ± sd	138.1 ± 90.3
Median (min-max)	128.0 (1.0–356.0)

Survival outcomes, *n* (%)	
Alive	78 (84.8)
Dead	14 (15.2)

**Table 2:** Overall survival rates according to factors.

	Log-rank test *p*	5-year survival rate (%)	10-year survival rate (%)
All patients		98.8	91.5

Sex	0.128		
Female		100	100
Male		97.9	84.8

Age	0.169		
≤30 years		100	97.4
>30 years		91.1	91.5

Race	0.179		
White		98.4	87.8
Black		100	100
Other		100	100

Marital status	0.481		
Single		97.8	89.9
Married		100	92.6

Laterality	0.169		
Left		97.1	89.4
Right		100	92.8

Diagnosis years	0.374		
1973–1997		100	84.6
≥1998		98.3	96.0

Surgical treatment	0.809		
No		100	100
Yes		98.6	91.5

In this study, median (95% CI) survival time could not be reached and therefore, mean and std. Error is presented.

## Data Availability

All data generated or analyzed during this study are included in the following site: https://seer.cancer.gov/. Requests for material should be made to the corresponding author.
